# Comments re article on comparison of performance and abnormal cell flagging of two automated hematology analyzers: Sysmex XN 3000 and Beckman Coulter DxH 800

**DOI:** 10.1111/ijlh.13132

**Published:** 2019-11-26

**Authors:** Elena Sukhacheva

**Affiliations:** ^1^ Medical and Scientific Affairs Beckman Coulter Eurocenter Nyon Switzerland

Dear Editors,

We read with interest the paper of Genc et al^1^ published in the *International Journal of Laboratory Hematology.* As per the paper's Abstract Introduction, “The purpose was to evaluate the analytical performances of Sysmex XN 3000 and UniCel DxH 800 comparing the obtained results with manual counting and between each other. Also flagging capabilities of abnormal cells were compared for both analyzers.”

Unfortunately, some statements in the Genc paper were non‐supported or incorrect, and important data were not discussed in the Conclusions. These issues are described here.

1. The Abstracts’ Results section states: “Within‐run and between‐day coefficient of variations (CV%) of XN 3000 for hemoglobin, RBC, MCV, WBC, and platelets were lower than 5% and for WBC differentials lower than 10% except monocytes, which was 15.6% at low level. The precision results of UniCel DxH 800 were also lower than 5.0% except platelets (9.5%) and monocytes (45%) at low level.” This statement is at odds with the data presented in the paper's table 1, and in fact, those data are instead consistent with all previous publications^2^, ^3^, ^4^ to date which demonstrate similar or better performance for UniCel DxH 800 PLT compared with Sysmex XN PLT. The presence of this statement in the Abstract is particularly problematic, as many readers will not read the full text nor inspect the data tables closely enough to discern the error.
2. In describing their method for defining positive smear findings, the authors cite Barnes et al^5^: “Morphologic criteria were used to define positive smear findings regarding blasts, NRBC, IGs, and ALs. The recommended threshold by the International Society for Laboratory Hematology is 1% for IGs, 4% or more for ALs, 0.5% for blasts, and 1% for NRBC.” The criteria listed are not consistent with those proposed by Barnes et al, wherein the thresholds are blast ≥1%, metamyelocyte >2%, and myelo/promyelocyte ≥1%.
3. The authors state, “According to our findings, the XN 3000 and DxH 800 are accurate, highly precise systems, which can operate effectively in high‐volume clinical laboratories with increased workflow. However, the XN 3000 analyzer seems to be more effective in detecting blasts, IGs, and ALs than the DxH 800. Only the NRBC results were similar for both analyzers. Detection of abnormal cells with high sensitivity may improve laboratory workflow with reduced slide review rates and accelerated turnaround times.” They conclude that XN 3000 is more efficient in detecting blasts, IGs, and ALs.

But according to their table 4, DxH 800 shows higher % efficiency for detecting blasts and ALs, respectively: 83.3 for DxH 800 vs 68.6 for XN and 84.3 for DxH 800 vs 62.7 for XN.

4. The Abstract's Conclusion states: “The detection of abnormal cells with high sensitivity may improve laboratory workflow with a reduced slide review and accelerated turnaround time.”

According to their table 4, here is the total number of false positives:

Sysmex: 27 + 19+5 + 31= 82;

Beckman: 9 + 6+5 + 6=26.

Sysmex has greater than three times the number of false positives as compared to Beckman Coulter. To conclude that reduced slide review and accelerated turnaround time would result in misleading. Again, inclusion of this statement in the Abstract is particularly problematic, as many readers will not inspect the data tables closely enough to discern the discrepancy.

5. Finally, according to the data presented in table 3, UniCel DxH 800 demonstrates better correlation with the reference method for Ly, Mono, and IG than Sysmex XN 3000, with Ly correlation coefficient of *r* = 0.8749 for DxH 800 vs *r* = 0.8310 for Sysmex XN 3000, Mono correlation coefficient of *r* = 0.4610 for DxH 800 vs *r* = 0.3398 for Sysmex XN 3000, and IG correlation coefficient of *r* = 0.9224 for DxH 800 vs *r* = 0.7431 for Sysmex XN 3000. Omission of these results from the discussion may mislead readers.

As Genc et al note, today's clinical laboratories benefit greatly from improved automated hematology systems and further point out that “evaluation of side‐by‐side performance of analyzers is essential to determine CBC and WBC differentiation, and their flagging capabilities in the presence of abnormal cells.” Findings from these evaluations must present accurately and reflect the data obtained in the study. Failure to do is misleading and may create incorrect perceptions about currently available analyzers.

## CONFLICT OF INTEREST

The author is Beckman Coulter employee.
